# Seroprevalence and risk factors associated with calves’ cryptosporidiosis in Egypt

**DOI:** 10.1186/s12917-025-05216-7

**Published:** 2026-01-27

**Authors:** Tamer Helmi Abd El-Aziz, Emad Beshir Ata, Mohamed Abdelmoghny Helal, Eman Hussein Abdel-Rahman, Soad Mohamed Nasr

**Affiliations:** Department of Parasitology and Animal Diseases, Veterinary Research Institute, National Research Centre, P.O. Box 12622, 33 Bohouth Street, Dokki, Giza, Egypt

**Keywords:** Calves cryptosporidiosis, Epidemiology, Clinical signs, rCp23, ELISA, Risk factors, Biochemical analysis

## Abstract

Cryptosporidiosis is a common worldwide cause of diarrhea in livestock. This study aimed to investigate the seroprevalence of cryptosporidiosis in calves, identify the related risk factors and determine certain serum biochemical parameters. A clinical examination was conducted on a total of 184 diarrheic and apparently healthy calves located in 3 Egyptian governorates, followed by the collection of blood and faecal samples. The Cp23 protein-coding gene was cloned into the pGEX-4T-1 vector followed by transfection and expression.The obtained purified protein was used to develop an ELISA for serodiagnosis. Serum protein and electrolyte profiles were measured. A structured questionnaire was used for risk factor assessment. The most prominent sign was watery or soft diarrhea although some animals were normal. Successful recombination, protein expression and characterization were confirmed by Western blotting. The infection rate was 26.1 ± 6.4% (19.7–32.4%) and 56.5 ± 7.16% (49.3–63.7%) by mZN and rCp23-ELISA, respectively. The relevant risk factors revealed that age, gender, housing type, bedding type, and faecal consistency were the most significant related risk factors. The biochemical analyses showed the presence of hyponatremia, hypokalemia, and hypochloremia in the infected calves with elevation in total proteins and globulins levels. Finally, the current study clarifiedthe high prevalence of cryptosporidiosis in the diarrheic and non-diarrheic calves that should be controlled through establishing multiple structured surveys in variant areas of the country. Preventive measures via dam vaccination, optimum colostrum intake, prophylactic treatment of the newly born calves, and good management of the farming system become mandatory.

**Supplementary Information:**

The online version contains supplementary material available at 10.1186/s12917-025-05216-7.

## Introduction

The farm animals play an important role in keeping of global food security [1–3]; they subjected to different exposed frequently to various pathogens that affect their health status and productivity [4–7].

Cryptosporidiosis represents one of the most commonly distributed diseases which cause diarrhea in a wide variety of hosts. The disease is caused by *Cryptosporidium* spp., an obligate intracellular protozoan infects the gastrointestinal tract which resulting in a great significant impact especially in immunodeficient cases and emerged as a life-threatening agent [8, 9]. Additionally, the high susceptibility of wide range of animal species to infection is considered to be apotential reservoirfor human cryptosporidiosis [10].

The construction of active surveys is considered the most important effective approach for efficient disease diagnosis and the establishment of effective control strategies for many diseases [11, 12]. The prevalence of cryptosporidiosis varied between countries ranging from 13.8% in Ethiopia [13], to 91.6% in Slovakia [9]. This wide range of variability depends on different risk factors including mainly age, animal breed, hygienic conditions, administration of colostrum, and immune status of the infected host [14, 15].

Acid-fast stain including modified Ziehl–Neelsen (mZN) is the most extensively used technique for the detection of *Cryptosporidium* oocysts, but it is time-consuming and subjective [16]. Besides, this method might not estimate all past infections due to the short duration of oocyst shedding, and the number of oocysts may be below the test’s detection limit [17]. The enzyme-linked immunosorbent assay (ELISA)-based technique for *Cryptosporidium* antibody detection provides a rapid, simple, and more sensitive alternative approach to conventional staining methods [18].

The sporozoite and merozoite surface proteins of *Cryptosporidium* are the main antigens that elicit protective immune responses [19]. Among these antigens, Cp23 is considered immunodominant and the most promising marker of infection since it is capable of inducing recognizable serum antibodies in humans and many animal species [20].

This study aimed to clone and express the Cp23 as glutathione s-transferase (GST)-fusion protein for accurate serodiagnosis of calves’ cryptosporidiosis using indirect ELISA in Egyptian Governorates; Sharqia, Ismailia, and Giza, andstudyingits related risk factors. Furthermore, serum proteins and electrolytes’ profiles were also determined.

## Materials and methods

### Ethics approval

This study was approved by the Ethical Committee for Medical Research (ECMR) at National Research Centre (NRC), Egypt. Protocol (Approval No. 051451123 on November 28, 2023). Informed consent was obtained from all animal owners prior to sample collection. All experimental procedures, including collection of blood and faecal samples were conducted in accordance with institutional Animal Welfare guidelines.

### Sampling areas and animal clinical examination

A total number of 184 diarrheic and non-diarrheic calves found at private farms and small holders located at Sharqia (30.7°N, 31.63°E), Ismailia (30.5831° N, 32.2654° E.), and Giza (28.7666° N, 29.2321° E) Governorates, Egypt were used in this study. All the tested animals were of cattle species with an agerange from 1 to 10 weeks. The cases were clinically examined [21]. The faecal smears were prepared, stained by mZN, and examined under oil immersion at 100X magnification. The positive and negative faecal samples were determined in terms of the presence or absence of *Cryptosporidium* oocysts in the stained faecal smears. The blood samples were collected on plain vacutainer tubes (without anticoagulant), centrifuged at 400 xg for serum separation, and storedat − 20 °C for further analyses.

### Questionnaire

All data of the animals examined in this study were collected during samples’ collection through a detailed questionnaire based on the previously published data according to [15] which was filled out through interview with the farms’ owners and/or the records. These data were either related to the host like the breed, age, sex, body condition score, faecal consistency, and curing time. Also, management factors including farm type, colostrum administration, history of infection, hygienic conditions, bedding types, and source of water were included.

### Parasite

Oocysts of *Cryptosporidium* spp.in this study was isolated from a naturally infected neonatal Holstein–Friesian calf suffering from diarrhea. The oocysts were identified by the mZN staining [22], then purified by discontinuous sucrose gradients [23], counted using a hemocytometer, diluted with saline to obtain 10^6^ oocysts/ml and stored at −80 °C.

### Amplification and cloning of Cp23 gene

The genomic DNA was extracted from purified oocysts of *Cryptosporidium* using the GeneJET Genomic DNA Purification Kit (Thermofisher)^®^. Partial amplification of the Cp23 gene was carried out using a designed specific forward primer (5´-acgcGGATCCATGGGTTGTTCATCATCAAAGC-3´) and reverse primer (5´-cattGAATTCTTAGGCATCAGCTGGCTTG-3´) (The underlined, bold uppercase letters are the sequence of the restriction enzymes) based on the Gene-Bank sequence (U34390). The thermal program included 1 cycle of initial denaturation at 94 °C for 10 min., 5 cycles consisting of denaturation at 94 °C for 30 s., annealing at 55 °C for 30 s, and extension at 72 °C for 30 s., 25 cycles consisting of denaturation at 94 °C for 30 s., annealing at 58 °C for 30 s, and extension at 72 °C for 30 s., 1 cycle of final extension at 72 °C for 7 min.

**Fig. 1:**
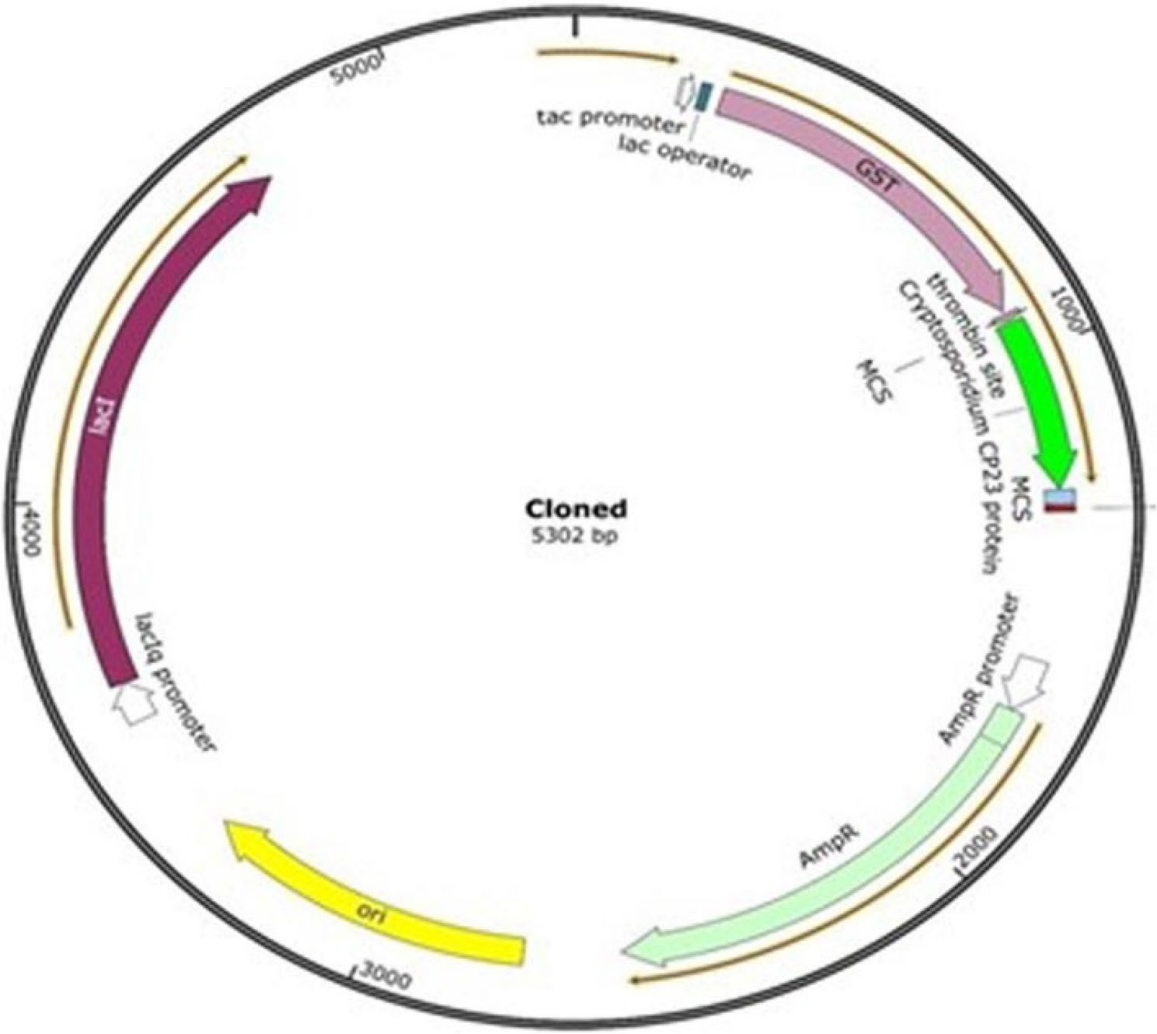
Schematic diagram shows that the nucleotide map of the cloned pGEX 4 T-1 vector with the amplified *Cryptosporidium* Cp23. Also, the amplified gene was inserted and confirmed to be in-frame with the GST-tagged protein and the thrombin site

The purified amplicons were digested using the *BamH1* and *EcoR1* restriction enzymes (Thermofisher scientific) followed by ligation with the pGEX 4-T-1 expression vector (GE Healthcare, USA) using the T4 DNA ligase enzyme (Thermofisher scientific) according to the manufacturing’s instruction as shown in Fig. 1. Transformation of the BL21 competent cells was done according to GST Gene Fusion System Handbook. The cloned vector was extracted using the GeneJET Plasmid Miniprep Kit (Thermofisher scientific). The right orientation was confirmed by sequencing of the extracted vector using the vector specific pGEX 5’ Sequencing Primer (5´-GGGCTGGCAAGCCACGTTTGGTG-3´), and pGEX 3’ Sequencing Primer (5´-CCGGGAGCTGCATGTGTCAGAGG-3´). The thermal program included 1 cycle of initial denaturation at 94 °C for 10 min., 30 cycles consisting of denaturation at 94 °C for 30 s., annealing at 58 °C for 30 s, and extension at 72 °C for 30 s., 1 cycle of final extension at 72 °C for 7 min.

### Expression and purification of recombinant antigen (rCp23)

To produce the GST-Cp23 fusion protein, the transformed *Escherichia coli* (*E.coli*) (BL21) cells were cultured in Luria–Bertani (LB) medium (Oxoid, UK) supplemented with Ampicillin as the selective antibiotic. Protein expression was induced by adding isopropyl β-D-1-thiogalactopyranoside (IPTG) with multiple concentrations including 0.1 mM, 0.5 mM, 1mM and incubation at different temperatures including 37 °C for 8 h, 25 °C for 10 h, and 16 °C for overnight with continuous shaking. The bacterial pellets were collected by centrifugation at 12,000 xg for 10 min at 4 °C and suspended in ice-cold PBS followed by sonication (30-sec pulse, 30-sec pause for 10 min).The obtained supernatant was collected and used for the subsequent purification using the Glutathione Sepharose™ 4B resin (GE Healthcare, USA) according to the manufacturer’s instructions. The eluted recombinant protein was dialyzed in PBS (0.5 M, pH 7.2) for 3 days. Finally, the concentration of the purified rCp23 protein was determined using the method of Lowry et al. [24].

### Electrophoretic characterization of the expressed and purified protein

The expression and purification processes of Cp23 protein were monitored by electrophoretic separation of subsequent 40 µg protein samples under reducing conditions on 12% Tris-Glycine SDS-PAGE using vertical electrophoresis apparatus (SCIE-PLAS, Cambridge, UK) according to the method ofLaemmli [25]. The resultant electrophoretic band patterns were developed by fixing the gel with a mixture of acetic acid: methanol: double-distilled water (1:4:5), followed by staining with 0.1% Coomassie blue dye and destained in the fixation mixture. The gel was then imaged and analyzed by the Gel DoCTM XR System (Bio-Rad, USA). The relative molecular weights of the separated protein bands were calculated based on molecular weight standards of the pre-stained protein ladder which was electrophoresed on the same gel.

### Immunoblotting of the purified recombinant Cp23 protein

Western blotting was utilized to identify the immunoreactivity of the purified recombinant Cp23 protein against naturally infected bovine serum with *Cryptosporidium* spp. The protein bands were electrophoretically transferred from SDS-PAGE to a nitrocellulose membrane following the method of Towbin et al. [26]. The sheet was then blocked overnight with 1% dry skimmed milk in Tris-Buffered Saline TBS, followed by extensive washing in TBS and incubation for 1½ h with primary antibodies; negative control and naturally infected bovine serum with *Cryptosporidium* spp. (1:100 diluted in 0.5% BSA/TBS). After three times washing, the sheet was incubated for 1½ h at room temperature with horse radish peroxidase-conjugated anti-bovine IgG (Sigma-Aldrich, USA) diluted1:1000 in 0.5% BSA/TBS as a secondary antibody. Finally, the sheet was rinsed again with the TBS washing buffer before being exposed to the substrate buffer (0.06% 4CN in 10 mL methanol, completed to 50 mL with TBS, pH 7.5 and 30 µl 30% H_2_O_2_) for 5–30 min. The developed reaction was stopped by rinsing the sheet in distilled water and subsequently dried, imaged, and analyzed in the Gel DoCTM System (Bio-Rad, USA).

### Enzyme-linked immunosorbent assay (ELISA)

The optimum condition for using the purified recombinant Cp23 protein in the diagnosis of bovine cryptosporidiosis by indirect ELISA was determined based on preliminary checker-board titrations [27]. The concentrations of coating antigen (1, 2 and 3 µg/mL), tested serum (1:100, 1:200 and 1:400), and conjugate (1:1000, 1:5000 and 1:10000) were tested for optimum condition using positive and negative control sera.The optimal concentrations were detected at 2 µg/mL, 1:100 and 1:1000 for antigen, serum and conjugate, respectively. Briefly, the purified recombinant Cp23 protein (2 µg/mL diluted in carbonate-bicarbonate coating buffer, pH 9.6) was coated on a 96-well plate and incubated for 1 h at 37 °C, then kept overnight at 4 °C. The next day, the plates were washed three times with PBS-Tween 20 (0.05%) and then blocked with the blocking buffer (2% dry skimmed milk in coating buffer) (200 µl/well) for 1 h at room temperature. After that, the plates were extensively washed, and the bovine serum samples (1:100 dilution in 0.1% dry skimmed milk/PBS-T) were added in triplicates and left for 1½ h at 37 °C. The plates were washed again three times with PBS-T followed by incubation with 100 µl of peroxidase-conjugated anti-bovine IgG at a 1:1000 dilution for 1½ h at 37 °C. After further washing, the colorimetric reaction was developed by adding 100 µl/well of substrate buffer (0.04% OPD in 50 ml citrate/phosphate buffer, pH 5 and 20 µl of 30% H_2_O_2_) within 10–20 min at 37 °C.The reaction stopped with a solution of 0.16 M sulfuric acid. The optical densities (OD) values were measured in a microplate reader at 405 nm wavelength. All the assays were performed independently three times and inthree technical replicates.

A total of 12 positive control sera were collected from calves that had experienced cryptosporidiosis by mZN faecal examination. The negative control sera consisted of 6 samples collected from calves before receiving colostrum. The negativity of samples was laboratory-checked by three consecutive mZN faecal examinations.The cut-off value was calculated as the mean OD plus three times of the standard deviations of negative control sera [28]. Furthermore, reference sera from calves infected with *Toxoplasma gondii*, were also analyzed to assess cross-reactivity.

### Serum biochemical analyses

The serum levels of the total proteins [29], albumin [30], sodium [31], potassium [32], chloride [33], and calcium [34], were measured spectrophotometrically using the specific kits purchased from Centronic GmbH, Germany. Total globulins were estimated by subtracting the obtained value of albumin from the total proteins.The albumin/globulins (A/G) ratio was also calculated.

### Statistical analyses

Statistical Package for the Social Sciences (SPSS) program (version 20.0) at (*P* < 0.05) was used for the statistical analysis.The normality and homogeneity of data distribution was assessed using the Shapiro-Wilk test and Levene’test, respectively.The normally distributed data of serum biochemical analyses were expressed as mean ± standard error and the statistical analyses were performed by one-way analysis of variance (ANOVA). Tukey’s HSD post-hoc test was applied for pairwise comparisons with correction for multiple testing. Significant differences of infection rate and the related risk factors of the tested animals were estimated through the chi-squared test (χ^2^) when at least 80% of expected cell frequencies were ˃5. Logistic regression was performed for Cp23-ELISA seropositivity as the primary outcome variable to examine the association between infection and different risk factors. Odds ratios with 95% confidence intervals (CI) were calculated [35].

## Results

### Clinical signs

**Fig. 2:**
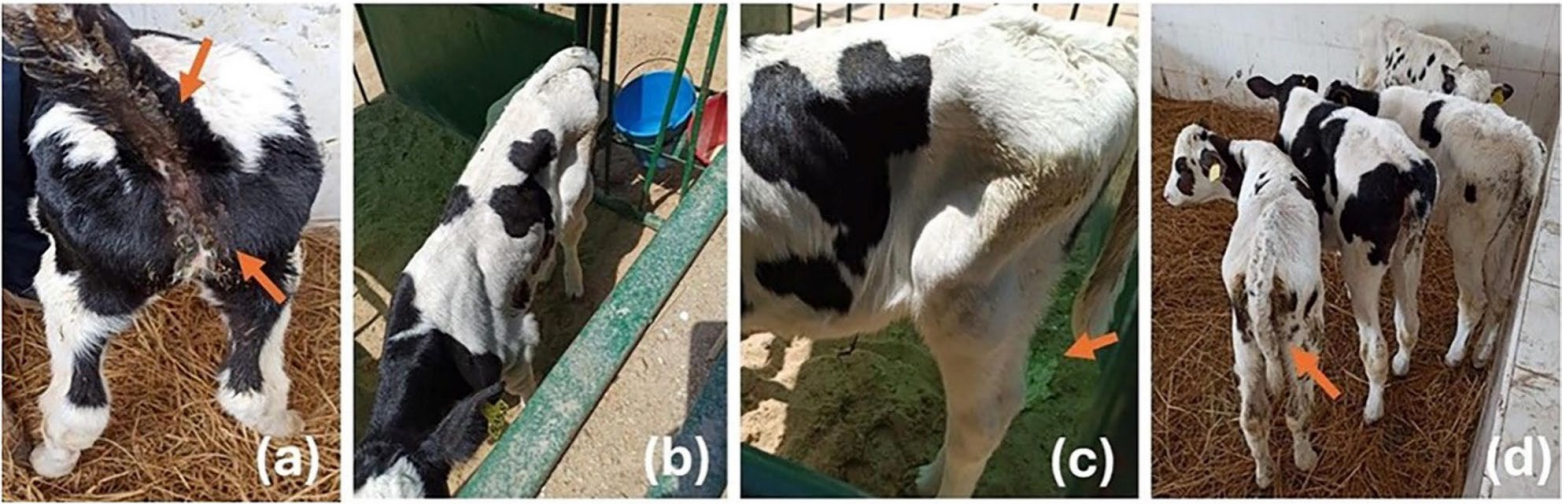
The figure shows some of the clinical features, and the housing conditions recorded during clinical examination of the tested animals (**a**) Calf suffered from diarrhea that stained the perineal area and tail region, (**b**) Apparently health calf found in individual box with sand ground, (**c**) Calf found in individual box with sand ground having soft feces, and (**d**) Mixed calves suffer from diarrhea or apparently health found in shared pens with a bedding ground

The clinical findings of cryptosporidiosis in examined calves showed mild to severe diarrhea and in some cases characterized by profuse and watery diarrhea, depression, weakness, loss of appetite with different degrees of dehydration, weight loss and emaciation. Some infected animals were clinically normal. Most of the infected animals were found in farms with a historical infection. However, non-infected calves were found to be healthy with no prominent clinical signs. (Fig. 2)

### Amplification and cloning of the Cp23

The extracted genomic DNA of *Cryptosporidium* spp. was used as a template. Using the designed primers resulted in successful partial amplification of the specified 336 bp of the Cp23 coding gene. Successful cloning was confirmed by colony PCR using the designed vector primers. Analysis of the sequencing results using the free online BLAST tool (https://blast.ncbi.nlm.nih.gov/Blast.cgi) confirmed the identification and correct orientation of the cloned gene. The obtained nucleotide sequence has been uploaded to GenBank under accession number OM417336.1.

### Expression, purification and immunoblotting of Cp23 protein

Although the different expression conditions were tested, the optimum conditions were using (IPTG) with a final concentration of 0.1 mM, and incubation at 37 °C for 8 h.

**Fig. 3:**
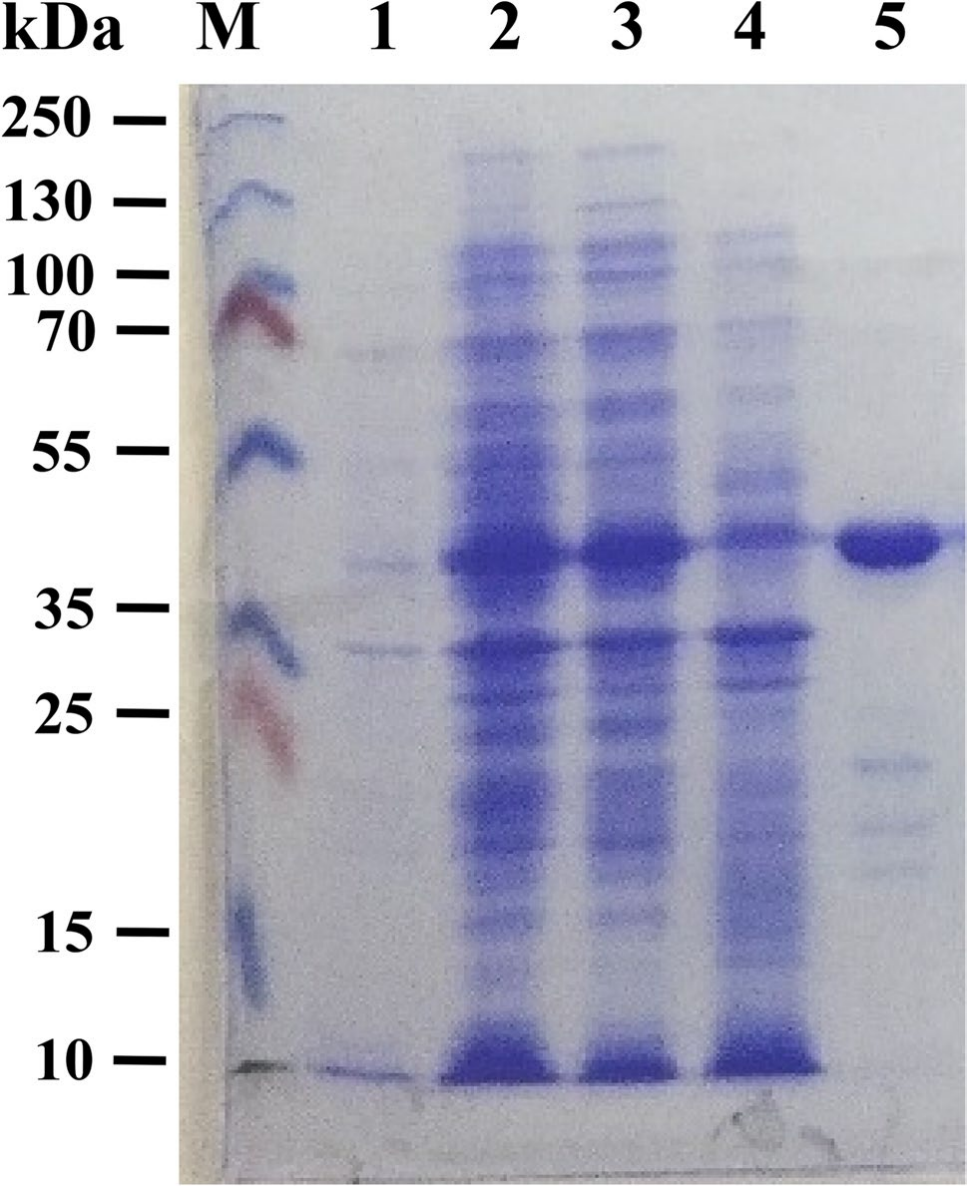
SDS-PAGE gel of the purified Cp23 antigen. Lane M: molecular weight marker. Lane 1: *E. coli* (BL21) bacterial culture in the absence of IPTG. Lane 2: *E. coli* (BL21) bacterial culture in the presence of 0.1 mM IPTG; Lane 3: *E. coli* (BL21) bacterial lysate supernatant and Lane 4: *E. coli* (BL21) bacterial pellets debris; Lane 5: purified Cp23 antigen with approximately 46 kDa

After expression, the GST/Cp23 fusion protein was purified and the immunogenic reaction was analyzed by SDS-PAGE, which showed the expected 46 kDa (Fig. 3).

**Fig. 4:**
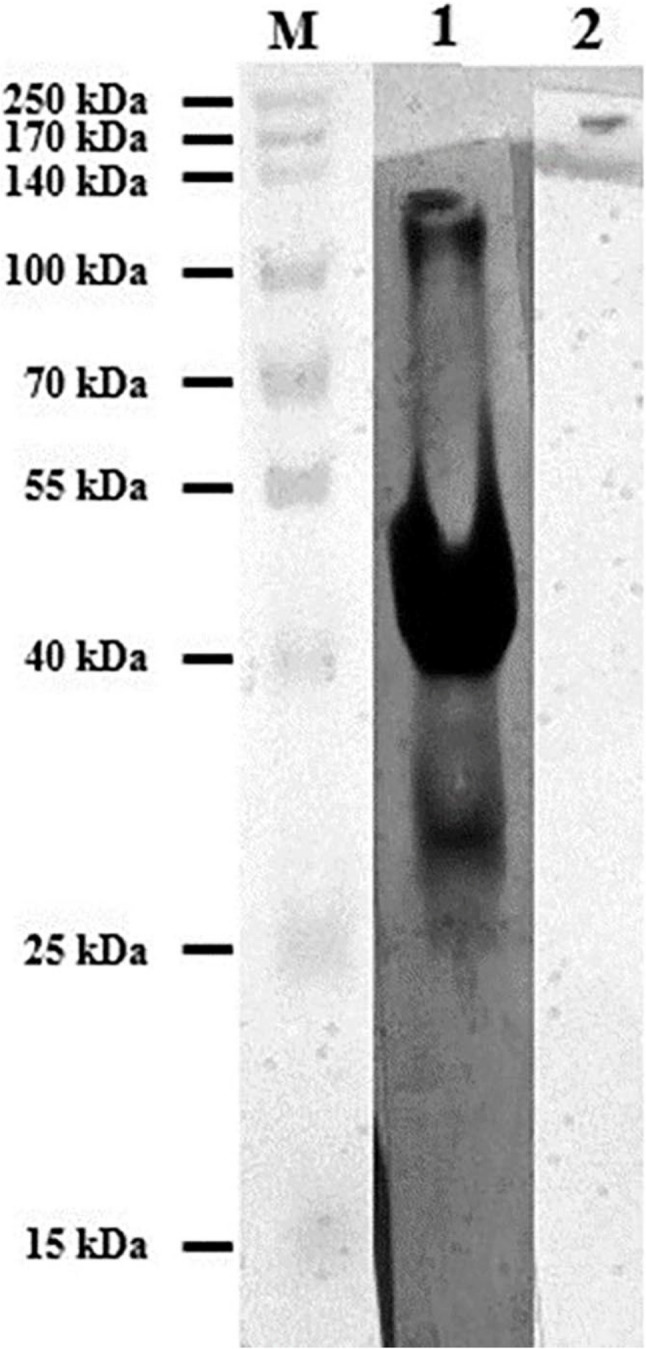
Western Blot analysis showing reactivity of GST-Cp 23 recombinant antigen against different sera. Lane 1: Prestained molecular weights protein marker, Lane 2: Naturally infected calf serum with Cryptosporidium Spp. and Lane 2: Control negative calf serum

The 46 kDa fusion protein gave a strong reaction with antibodies of naturally infected calfs erum as shown in Fig. 4. Moreover, there was no cross-reactivity between the recombinant Cp23 antigen and positive bovine sera for *Toxoplasma gondii*.

### Cryptosporidiosis infection rate

Using the mZN, only 48 out of the 184 examined calves (26.1 ± 6.4%) were positive for cryptosporidiosis. Assessment of the collected serum samples for the presence of IgG against *Cryptosporidium* spp. using the developed ELISA showed an infection rate of 56.5 ± 7.16% (104/184) of samples. All related data were explored in Table 1.

**Table 1:** The comparison between mZN and Cp23-ELISA assay in the detection of *Cryptosporidium* in naturally infected calves. (*N* = 184)

mZN/Cp23-ELISA	No. of animals	Percentages
-/-	64	34.8%
+/-	16	8.7%
-/+	72	39.1%
+/+	32	17.4%
Total number of positive results		
mZN	48	26.1%
Cp23-ELISA	104	56.5%

*mZN* Modified Ziehl–Neelsen, *ELISA * Enzyme-linked immunosorbent assay

(-/-)= (mZN/Cp23-ELISA)-negative calves

(+/-)= (mZN-positive/Cp23-ELISA-negative) calves

(-/+)= (mZN-negative/Cp23-ELISA-positive) calves

(+/+)= (mZN/Cp23-ELISA)-positive calves

#### Risk factors analysis

The different risk factors were evaluated and statistically analyzed (Table 2). The infection rate in the Friesian calves was (61.9%, 70/113) which was higher than that recorded in the cross-breed animals (47.8%, 34/71) with no significant difference (χ^2^ = 3.502, *P* = 0.386).

**Table 2:** Seroprevalence of cryptosporidiosis in calves from Egyptian governorates with different variables

Factors		Examined animals	Infected animals		χ^2^	Adjusted OddsRatios (95% CI)		*P*-value
			Positive	%				
Farm_ID	Farm_1	110	51	63.8	3.683	Ref		0.701 0.705
	Farm_2	45	21	26.3		1.22 (0.44–3.39)		
	Farm_3	29	8	10.0		0.79 (0.22–2.76)		
Breed	Friesian	113	70	61.9	3.502	1.43 (0.64–3.21)		0.386
	Cross	71	34	47.8				
Gender	Male	140	77	55	0.556	2.30 (1.06–8.50)		0.039
	Female	44	27	61.3				
Age	< 3 weeks	68	21	30.8	31.013	Ref		< 0.001
	3–6 weeks	42	27	64.2		0.08 (0.02–0.26)		
	6–10 weeks	74	56	75.6		0.07 (0.02–0.19)		
Farm type	Dairy	124	69	55.6	0.119	0.69 (0.29–1.65)		0.406
	Beef	60	35	58.3				
Colostrum administration	Natural suckling	99	48	48.5	5.678	0.96 (0.42–2.19)		0.920
	hand-or bottle-fed	85	56	65.8				
History of farm infection	Yes	76	40	52.6	0.796	0.50 (0.21–1.82)		0.114
	No	108	64	59.3				
Housing type	Individual boxes	34	8	23.5	18.813	0.07 (0.02–0.27)		< 0.001
	shared pens	150	96	64				
Hygiene condition	Good	82	39	47.5	4.842	1.55 (0.58–4.18)		0.387
	bad	102	65	63.7				
Bedding type	Hay	103	68	66	8.624	2.59 (1.10–6.13)		0.030
	Dust or sand	81	36	44.4				
Body condition scores	Good	85	41	48.2	4.424	1.25 (0.54–2.89)		0.606
	poor	99	63	63.6				
Faecal consistency	Normal	122	80	65.5	16.416	Ref		< 0.001 0.445
	Soft	36	10	27.7		7.21 (2.39–21.82)		
	Watery	26	14	53.8		1.62 (0.47–5.53)		
Total		184	104	56.5				

On the other side, statistical significance was recorded between the infection rate in females (61.3%, 27/44) compared to males (55%, 77/140) (χ^2^ = 0.556, *P* < 0.039).

Analysis of the rate based on the animals’ age revealed the presence of high significance (χ^2^ = 31.013, *P* < 0.001) between the group aged under 3 weeks (30.8%), and the other 2 groups ranged between 3 and 6 Weeks (64.2%) and the 6–10 weeks (75.6%).

Although the infection rate in dairy animals, (55.6%, 69/124) was lower than the beef types (58.3%, 35/60). But this difference was found to be non-significant (χ^2^ *=* 0.119, *P* = 0.406).

Colostrum administration was recorded in all tested calves either through natural suckling from their dams which have an infection rate of (48.4%, 48/99) which was insignificantly (χ^2^ = 5.678, *P* = 0.920) less than those having hand- or bottle-fed (68.8%, 56/85).

An infection rate of (59.2%, 64/108) was recorded in herds with no previous history which is insignificantly (*χ*^*2*^ = 0.796, *P* = 0.114) higher than those having a previous infection (52.6%, 40/76).

The housing type was found to be one of the most significant factors as the infection rate in calves reared in shared pens was (64%, 96/150). While the calves reared in individual boxes had an infection rate of (23.5%, 8/34) with a significant difference *(χ*^*2*^ = 18.813, *P* < 0.001).

The animals reared in good hygienic conditions had a lower infection rate of (47.5%, 39/82) compared to the animals in bad hygienic conditions (63.7%, 65/102) with no significant difference *(χ*^*2*^ = 4.842, *P* = 0.387). The animals that had hay as a bedding type were found to be significantly *(χ*^*2*^ = 8.624, *P* = 0.030) higher (66%, 68/103) than those in dust or sand (44.4%, 36/81).

The infection rate in the calves with good body condition (48.2%, 41/85) was lower than the poor condition calves (63.6%, 63/99) with no significant difference (*χ*^*2*^ = 4.424, *P* = 0.606).

Analysis of the infection rate in relation to faecal consistency revealed the presence of a high significance (χ^2^ = 16.416, *P* < 0.001) in the animals with a normal faecal nature (65.5%, 80/122) and the group with soft feces (27.7%), not those with the watery nature (53.8%).

### Biochemical serum analyses

Table 3 presents the results of serum biochemical analyses in calves classified according to parasitological (modified Ziehl-Neelsen staining) and serological (Cp23-ELISA) diagnostic status for *Cryptosporidium* infection. In the seropositive calves (‒mZN/+ELISA and + mZN/+ELISA), the results showed that the levels of serum total proteins were significantly increased as compared to serologically negative calves (‒mZN/‒ELISA and + mZN/‒ELISA), which was attributed to a significant elevation in total globulins. The level of serum albumin was markedly decreased in seropositive and non-oocyst-shedding calves (‒mZN/+ELISA) compared to the other groups. Serum sodium concentration was significantly decreased in parasitologically and serologically positive calves (+ mZN/+ELISA) as compared to negative calves (‒mZN/‒ELISA). In seropositive and oocyst-shedding calves (+ mZN/+ELISA), the serum potassium concentration was significantly decreased, while the serum chloride concentration was significantly increased compared to the oocyst-shedding and seronegative group (+ mZN/‒ELISA). In all groups of calves, no significant difference was recorded in serum calcium concentrations.

**Table 3:** Serum biochemical parameters in different groups of *Cryptosporidium*-infected calves diagnosed serologically (Cp23-ELISA) and parasitologically (mZN). (Mean ± SE, *N* = 5)

Parameters	(mZN/Cp23-ELISA)				Ƞ^2^	*P* value
	(-/-)	(-/+)	(+/-)	(+/+)		
Total proteins (mg/dl)	6.36 ± 0.20^**a**^	8.16 ± 0.14^**b**^	6.99 ± 0.12^**a**^	8.30 ± 0.21^**b**^	0.82	< 0.001
Albumin (mg/dl)	2.57 ± 0.14^**ab**^	2.90 ± 0.05^**b**^	2.63 ± 0.03^**b**^	2.43 ± 0.15^**a**^	0.59	0.038
Total globulins (mg/dl)	3.79 ± 0.06^**a**^	5.26 ± 0.16^**b**^	4.36 ± 0.14^**a**^	5.87 ± 0.35^**a**^	0.81	< 0.001
Albumin/Globulins ratio	0.68 ± 0.02^a^	0.55 ± 0.02 ^**a**^	0.61 ± 0.02^**ab**^	0.42 ± 0.05^**a**^	0.42	< 0.001
Sodium (mEq/l)	150.76 ± 1.69^**b**^	141.85 ± 0.79^**a**^	137.61 ± 1.53^**a**^	137.66 ± 0.24^**a**^	0.79	< 0.001
Potassium (mEq/l)	4.46 ± 0.22^**ab**^	4.57 ± 0.16^**ab**^	4.76 ± 0.03^**b**^	4.02 ± 0.04^**a**^	0.13	0.012
Chloride (mEq/l)	88.86 ± 0.72^**b**^	89.78 ± 0.26^**b**^	83.79 ± 0.81^**a**^	90.09 ± 0.04^**b**^	0.71	< 0.001
Calcium (mg/dl)	13.08 ± 0.58^**a**^	13.48 ± 0.85^**a**^	13.27 ± 0.49^**a**^	12.11 ± 0.37^**a**^	0.04	0.408

Means with different superscripts in the same row are significantly different at *P* < 0.05

*SE* Standard error, *mZN * Modified Ziehl–Neelsen, *ELISA * Enzyme-linked immunosorbent assay, Ƞ^2^ Eta squared

(-/-) = (mZN/Cp23-ELISA)-negative group. (-/+) = (mZN-negative/Cp23-ELISA-positive) group

(+/-)= (mZN-positive/Cp23-ELISA-negative) group. (+/+) = (mZN/Cp23-ELISA)-positive group

## Discussion

Infection with *Cryptosporidium* spp. is one of the leading causes of diarrhea in newborn calves. The most prominent signs of cryptosporidiosis in this study were diarrhea with different degree of dehydration, and weight loss [15].

To overcome the limitation of the staining technique in the current study, an indirect ELISA was developed based on production of rCp23 antigen and used for the serological diagnosis of infection which has the advantage of being more sensitive and specific than mZN [9].

Previous reports confirmed that prokaryotic GST was not serologically recognized by *Cryptosporidium*-infected sera [18]. So, the recombinant antigen Cp23 was synthesized to detect the antibodies against *Cryptosporidium* in naturally infected calves’ sera. However, the expected 46 kDa fusion protein was visualized by SDS-PAGE and gave strong reactivity in the immunoblot with serum of *Cryptosporidium*-infected calf but degradation bands were also detected as previously recorded by Iochmannet al. [36] who showed that 25 kDa and 28 kDa degradation bands were recognized by sera of *Cryptosporidium*-infected calf in the immunoblot technique and stated that these degradation bands did not affect the antigenicity of produced proteins.

In this study, faecal examination using mZN revealed that (26.1%) of animals were positive. Previous studies in different Governorates of Egypt using mZN for examining calf feces have reported different *Cryptosporidium* prevalence rates, ranging from 7.07% to 31.67% [37]. On the other hand, the investigation of calves’ sera by the developed rCp23-ELISA showed a high infection rate of 56.5%. A similar result was previously reported in an epidemiological study in Argentina [14].

Using rCp23-based ELISA in previous studies indicated that the seroprevalence in cattle sera was 35.9% in Egypt [38] and 42.7% in Japan [18]. On the other hand, a comparatively low infection rate was recorded in multiple studies previously conducted in Egypt (18.8%) [15] and Ethiopia (13.8%) [13].

Although the animal breed was found to be a non-significant factor, the infection rate in the Friesian calves was higher (61.9%) compared to the cross-breed ones (47.8%). Our results agreed with the previous recorded data that exogenous breeds were found to be more susceptible for having the infection compared to the local or the mixed ones [39]. Also, the ‘pure-bred’ animals were at higher risk than cross-breeds [40]. Conversely, a study concluded that the mixed breed had a high positive rate (21.54%) compared to the Holstein one (14.71%) [15].

In the current study, sex was found to be a significant factor for infection occurrence as high antibodies ratio was determined in female calves compared to the male ones. The same results were previously recorded [41]. On the other hand, different studies found that sex had no effect on the prevalence of cryptosporidiosis [15, 42].

The high prevalence was determined in calves more than 3 weeks old compared to the younger ones. The specific serum antibodies response to enteric cryptosporidiosis in cattle was detected 2–3 weeks after infection [43]. These results were previously attributed to the immature immune system of young animals and resistance to infection might develop with age [44]. Although previous studies revealed that the high rate was more in the younger ages [45], with a peak at 9–14 days but this is because they detected the causative agent not the provoked antibodies. The presence of reverse correlation between the detection of specific IgG and the shedding of *Cryptosporidium* oocyst in young calves can be attributed to the relatively short duration of oocysts excretion and the latent period required for antigenic stimulation to produce specific antibodies.

The percentage of infection in dairy farms was higher than the beef ones with no significance. Previously, a higher rate (79.41%) was reported in young dairy calves in Punjab [46].

Although all examined calves were confirmed to have colostrum, the infection rate was insignificantly higher in those having hand- or bottle-fed rather than the natural suckling ones. These data match with a previous study which concluded that bottle feeding was riskier with no significant difference [47]. While others found that, bottle feeding had no impact on infection risk [48].

The obtained data cleared that the presence of farm case history did not significantly affect the infection rate. However, it was higher in the animals from farms with no previous history which agree with a previous study [48]. Meanwhile, an earlier one cleared that the presence of a prior history of herd infection was positively associated with high shedding of *C. parvum* [49].

Animals reared in individual boxes were found to be significantly less infected than those in the shared or group pens as previously recorded [50]. Although other studies proved that the presence of individual boxes did not affect the disease risk [51]. On the other side, housing of calves in single pens was found to be associated with a higher prevalence percentage than housing them in individually outdoors [52].

Bad hygiene conditions were found to be highly associated with the occurrence of infection. The same results were previously reported [15]. This phenomenon is related to the bedding type and persistence of the oocysts in a muddy environment.

Bedding type was found to significantly affect the infection rate. The percentage of the infected animals reared on hay was higher than those on dust or sand. The obtained data was aligned with [53]. Other studies concluded the presence of higher rates on the different bedding types including dust, and shavings [51].

Although presence of watery diarrhea at young ages or even before weaning is a common criterion of cryptosporidiosis in most of the conducted studies [39]. But interestingly, a high percentage of the infected cases were found to have normal faecal consistency. This could be explained as many cases were confirmed to be infected although they did not exhibit clear asymptomatic or even post-mortem changes [54], especially infection with the *C. andersoni* type [55].

The infection rate was insignificantly, higher in the poor body condition calves compared with the good-conditioned ones. This observation was previously reported [13], which could be attributed to the chronic nature of the parasite or the farm’s poor management [44].

Concerning the biochemical analyses, in neonatal *Cryptosporidium*-infected calves, dehydration, and electrolyte imbalance are common complications of diarrhea. Therefore, hypokalemia, and hypochloremia are often present in calves that have diarrhea. However, in acute cases, calves may have elevated blood potassium concentrations [56]. This contradictory situation may be attributed to metabolic acidosis and increased exchange between hydrogen and potassium ions with reduced renal elimination of potassium [57]. It is important to note that the faecal samples in the present experiment were randomly collected from neonatal calves regardless of whether or not they had diarrheal symptoms, which may lead to inconsistency between the present results and previous ones. Hyponatremia, hypokalemia, and hypochloremia were observed in mZN- and Cp23-positive calves which may be attributed to excessive electrolyte loss, which is consistent with the result of Kang et al. [58]. Ha et al. [59] demonstrated that no significant changes were observed in the levels of sodium and chloride in *Cryptosporidium*-infected calves. However, the level of potassium was significantly elevated. For the protein profile, the present data showed a significant elevation in the levels of total proteins and globulins in *Cryptosporidium*-seropositive calves. These findings were consistent with the studies of Ha et al. [59] and Soufy et al. [60]. The significant increase in the level of serum total proteins may be related to the increase in the level of total globulins, which in turn increased as a consequence of the immune response against infection.

### Conclusion and recommendations

The current research elucidated successful amplification, cloning, expression, and purification of the *Cryptosporidium* Cp23 protein, which was used to develop an in-house ELISA for serodiagnosis of cryptosporidiosis in calves from Egypt. Age, colostrum administration, housing type, bedding type, and faecal consistency were the most significant related risk factors. The infected cases showed the presence of hyponatremia, hypokalemia, and hypochloremia with elevations in total proteins and globulin levels. The recorded high prevalence rate (56.5%) highlighted the endemicity of this disease. Accordingly, application of control measures for the newly born calves, especially those from non-vaccinated dams, is highly recommended. Application of high sanitary measures during and after parturition, colostrum administration at the optimum time, and optimum bedding environment become obligatory. Although this study had many advantageous aspects that help to understand this disease effectively, several limitations should be acknowledged. First, calves were sampled from only three farms, which precluded the use of mixed-effects models to generalize findings to the broader population of farms. Second, the comparatively limited number of tested animals may limit our prevalence estimates. Future studies involving larger numbers of farms across multiple governorates should be employed to confirm the prevalence ratio. Lastly, the application of molecular detection and identification is advisable.

## Supplementary Information

Supplementary Material 1.

## Data Availability

In this study, the generated sequence has been deposited in the GenBank database under accession number (OM417336.1) and the other data used in the present study are available from the corresponding author on reasonable request.
