# Supplementary material for: Seroprevalence and risk factors associated with calves’ cryptosporidiosis in Egypt
Source: BMC Vet Res. 2026 Jan 27;22:125. doi: 10.1186/s12917-025-05216-7 (PMC12930809; doi:10.1186/s12917-025-05216-7)
Supplement: Supplementary file 1 — Supplementary Material 1. [file 12917_2025_5216_MOESM1_ESM.docx]

| 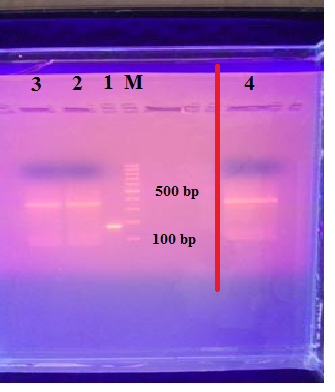 |
| --- |
| **Fig. 3** Gel electrophoresis of PCR products on 2% agarose stained with ethidium bromide. Lane M: 100-bp molecular weight ladder. Lane 1: amplification of 180 bp fragment of uncut pGEX vector. Lane 2 , Lane 3 and Lane 4: amplification of 336 bp fragments of *Cryptosporidium* Cp23 gene. Lane 4: amplification of 336 bp fragments of *Cryptosporidium* Cp23 gene, which was cropped from this gel and omitted. |

|  |
| --- |
|  |

**Fig. 4** SDS-PAGE gel of the purified Cp23 antigen. Lane M: molecular weight marker. Lane 1: *E. coli* (BL21) bacterial culture in the absence of IPTG. Lane 2: *E. coli* (BL21) bacterial culture in the presence of 0.1 mM IPTG; Lane 3: *E. coli* (BL21) bacterial lysate supernatant, Lane 4: *E. coli* (BL21) bacterial pellets debris; Lane 5: purified Cp23 antigen with approximately 46 kDa Lanes A, B, C, and D: different concentrations of purified Cp23 antigen (46 kDa) which were cropped from this gel and omitted.

|  |
| --- |
| **Fig. 5** Immunoblotting of GST-Cp23 recombinant antigen. Lane M: pre-stained protein marker. Lane 1: GST-Cp23 antigen probed with serum from a naturally-infected calf with Cryptosporidium spp. Lane 2: GST-Cp23 antigen probed with serum from a healthy calf. Lanes M, 1, and 2 represent the immunoblots that were prepared separately in test tubes prior to the addition of primary antibodies for technical purposes. Their images were captured together. |
